# Feasibility Study of Developing a Saline-Based Antiviral Nanoformulation Containing Lipid-Soluble EGCG: A Potential Nasal Drug to Treat Long COVID

**DOI:** 10.3390/v16020196

**Published:** 2024-01-27

**Authors:** Nicolette Frank, Douglas Dickinson, William Garcia, Yutao Liu, Hongfang Yu, Jingwen Cai, Sahaj Patel, Bo Yao, Xiaocui Jiang, Stephen Hsu

**Affiliations:** 1 Department of Oral Biology & Diagnostic Sciences, Augusta University, Augusta, GA 30912, USA; nicolettefrank6449@yahoo.com (N.F.); wigarcia@augusta.edu (W.G.); spatel33@augusta.edu (S.P.); 2 Camellix Research Laboratory, Augusta, GA 30912, USA; dougdickinson4357@gmail.com; 3 Department of Cellular Biology and Anatomy, Augusta University, Augusta, GA 30912, USA; yutliu@augusta.edu (Y.L.); hongyu@augusta.edu (H.Y.); jincai@augusta.edu (J.C.); 4 Changxing Sanju Biotech Co., Ltd., Hangzhou 310013, China; yaobotea@126.com (B.Y.); jxc20057011222@126.com (X.J.)

**Keywords:** COVID-19, Long COVID, EC16, EGCG-palmitate, nanoformulations

## Abstract

A recent estimate indicates that up to 23.7 million Americans suffer from long COVID, and approximately one million workers may be out of the workforce each day due to associated symptoms, leading to a USD 50 billion annual loss of salary. Post-COVID (Long COVID) neurologic symptoms are due to the initial robust replication of SARS-CoV-2 in the nasal neuroepithelial cells, leading to inflammation of the olfactory epithelium (OE) and the central nervous system (CNS), and the OE becoming a persistent infection site. Previously, our group showed that Epigallocatechin-3-gallate-palmitate (EC16) nanoformulations possess strong antiviral activity against human coronavirus, suggesting this green tea-derived compound in nanoparticle formulations could be developed as an intranasally delivered new drug to eliminate the persistent SARS-CoV-2 infection, leading to restored olfactory function and reduced inflammation in the CNS. The objective of the current study was to determine the compatibility of the nanoformulations with human nasal primary epithelial cells (HNpECs). Methods: Nanoparticle size was measured using the ZetaView Nanoparticle Tracking Analysis (NTA) system; contact antiviral activity was determined by TCID50 assay for cytopathic effect on MRC-5 cells; post-infection inhibition activity was determined in HNpECs; and cytotoxicity for these cells was determined using an MTT assay. The rapid inactivation of OC43 (a β-coronavirus) and 229E (α-coronavirus) viruses was further characterized by transmission electron microscopy. Results: A saline-based nanoformulation containing 0.1% *w/v* EC16 was able to inactivate 99.9999% β-coronavirus OC43 on direct contact within 1 min. After a 10-min incubation of infected HNpECs with a formulation containing drug-grade EC16 (EGCG-4′ mono-palmitate or EC16m), OC43 viral replication was inhibited by 99%. In addition, all nanoformulations tested for their effect on cell viability were comparable to normal saline, a regularly used nasal irrigation solution. A 1-min incubation of an EC16 nanoformulation with either OC43 or 229E showed an altered viral structure. Conclusion: Nanoformulations containing EC16 showed properties compatible with nasal application to rapidly inactivate SARS-CoV-2 residing in the olfactory mucosa and to reduce inflammation in the CNS, pending additional formulation and safety studies.

## 1. Introduction

According to the Centers for Disease Control and Prevention (CDC), one of the major neurologic Long COVID sequelae is anosmia, which has a significant negative impact on patients’ quality of life [1]. Recently published clinical data demonstrate that only 5% of patients with Long COVID anosmia fully recovered during the past 2 years, and up to 10.4% of all COVID patients were still symptomatic 18 months after their initial SARS-CoV-2 infection [2,3]. Currently, there is no drug or other method to minimize Long COVID-associated neurologic symptoms. Thus, there is an urgent need to develop an effective approach to treat neurologic symptoms associated with Long COVID, which has been shown to involve persistent SARS-CoV-2 infection in the olfactory epithelium and inflammation in the nervous system [4].

The green tea polyphenol Epigallocatechin-3-gallate (EGCG) has a wide spectrum of antiviral activity [5,6]. We have tested the antiviral activity of EGCG against SARS-CoV-2, with promising results [7]. In addition to this well-described antiviral effect, EGCG also has a widely reported anti-inflammatory activity, and it has also been shown to provide neuroprotective effects [8,9]. However, EGCG is a strong antioxidant and chemically unstable, and is therefore quickly self-oxidized (auto-oxidation) in aqueous solutions. For example, EGCG dissolved in phosphate buffer saline completely converts to unstable EGCG auto-oxidation products within 4 h at 37 °C [9]. Our group also confirmed that it is impossible to maintain EGCG’s stability in a liquid formulation [10]. EGCG also has a low bioavailability in humans, with a maximum attainable serum concentration of 0.57 nM [11,12]. To overcome the disadvantages of water-soluble EGCG, encapsulation of EGCG in nanoparticles was studied as an approach to treat atherosclerosis and cancer [13,14,15]. Oral administration of chitosan polyaspartic acid nanoparticles loaded with EGCG demonstrated increased efficacy against atherosclerosis compared to EGCG feeding [13]. EGCG loaded in melanin nanoparticles exhibits improved stability and antioxidant activity, as well as antibacterial activity [16]. Oral administration of EGCG nanoparticles (10 mg/kg/day 30 days) prevented memory loss, neuritic plaque and neurofibrillary tangles formation in an aluminum-induced rat model for Alzheimer’s disease [17] EGCG PEGylated-PLGA nanoparticles (162 nm in size) have shown promising effect for the treatment of temporal lobe epilepsy in a mouse model [18]. However, this EGCG nanoparticle formulation was administered by intraperitoneal injection at a 30 mg/kg body weight dose (equivalent to 1800 mg in a person weighing 60 kg) over a 3-month period, instead of employing a direct route to the central nervous system (CNS) [18]. In this case, a nose-to-brain (NTB) delivery method was not considered, possibly due to the water-soluble nature of EGCG in the nanoparticles, which may not be able to effectively enter the CNS. Therefore, using a lipid-soluble form of EGCG could increase the effectiveness of CNS delivery.

Recent studies indicate that nose-to-brain drug delivery (NTBDD) using nanoparticles of a lipid-soluble drug or lipid carrier is a promising method to increase the drug bioavailability with rapid action [19,20,21]. Specifically, NTBDD technology has the potential to treat neurologic disorders by decreasing reactive oxygen species with natural antioxidants [22]. We reported recently that lipid-soluble derivatives of EGCG (EC16, EC16m) in glycerol-based nanoformulations possess antiviral activities against human coronavirus [23]. This finding indicates nasal delivery could be used as a preferred route to administer lipid-soluble EGCG for inactivating SARS-CoV-2 in the olfactory epithelium and for neuroprotection. Based on the evidence from our findings, we hypothesize that EGCG-palmitate (EC16) and its pharmaceutical form EGCG-4′ mono-palmitate (EC16m), compounds with multiple mechanisms of antiviral activity, plus anti-inflammatory, antioxidant, and neuroprotective properties, have the potential to become a new nasal drug for the minimization of Long COVID-associated neurologic symptoms such as anosmia. The objective of the current feasibility study was to determine the antiviral activity and cytotoxicity of saline-based antiviral nanoformulations of lipid-soluble EGCG when used with human nasal primary epithelial cells (HNpECs).

**Scheme 1:**
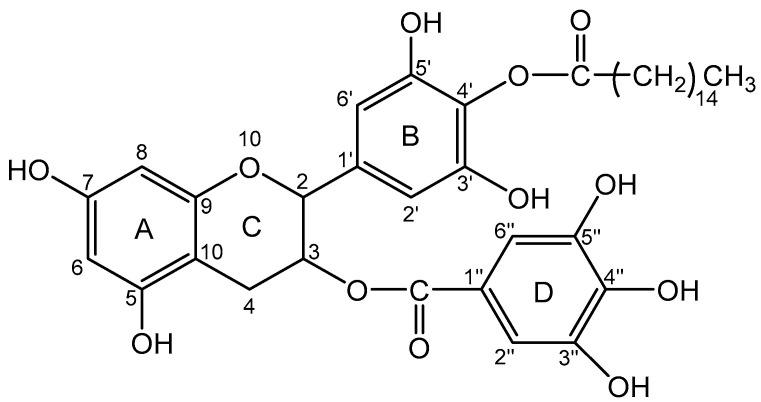
Schematic chemical structure of Epigallocatechin-3-gallate-4′ mono-palmitate (EC16m).

The only difference between EGCG and EC16m (Scheme 1) is that in EC16m the polyphenol is linked to a palmitoyl moety at 4′ carbon, converts the compound from water soluble to lipid soluble. The difference between EC16m and EC16 is that in EC16 in addition to the 4′ carbon the polyphenol is also linked to two or more palmitoyl moeties.

## 2. Materials and Methods

### 2.1. Virus and Cell Lines

OC43 human β-coronavirus (ATCC VR-1558), 229E human α-coronavirus (ATCC VR-740), MRC-5 human respiratory fibroblast cells (ATCC CCL-171), and HCT-8 human intestinal epithelial cells (ATCC CLL-244) were purchased from ATCC (Manassas, WV, USA). Human nasal primary epithelial cells were obtained from PromoCell (63/65 69,126 Heidelberg Germany). MRC-5 cells were used for antiviral assays for both OC43 and 229E cells. For viral propagation, HCT-8 cells were used for OC43, and MRC-5 cells for 229E, as recommended by ATCC.

### 2.2. EC16, EC16m, and Other Supplies

Epigallocatechin-3-gallate-palmitates (EC16, CAS# 1448315-04-5): a mixture of mono- di- and tri-palmitates) and Epigallocatechin-3-gallate-4′ mono-palmitate (EC16m, CAS# 507453-56-7), was provided by Camellix, LLC (Evans, GA, USA). Eagle’s Minimal Essential Medium (EMEM) was purchased from ATCC (30-2003). Fetal bovine serum (FBS) was from Neuromics (Edina, MN, USA). Trypsin (0.25%)-EDTA was provided by Fisher Scientific. Penicillin, streptomycin, and amphotericin B solution (100×) was obtained from Corning (Glendale, AR, USA). Plasticwares were purchased from Southern Labware (Cumming, GA, USA).

### 2.3. EC16 and EC16m Nanoformulations

EC16 (average formula weight 809) and EC16m (formula weight 697) were initially dispersed as stable glycerol-based stocks (F18 and F18m, respectively) at 1% *w/v* using a proprietary method. “F18” represents the 18th formulation method out of >100 nanoformulations methods attempted. “F18m” is the nanoformulation containing EGCG-4′mono-palmitate (EC16m). Working nanoformulations of 0.1% EC16 or EC16m were made by a 10-fold dilution with normal saline solution (0.9% NaCl), equal to approximately 1.25 mM (EC16) and 1.40 mM (EC16m), respectively. These were then diluted to lower concentrations for this study. To further improve the antiviral activity of the nanoformulations, a food-grade dispersing agent (proprietary information) was incorporated into the working F18 saline nanosuspension; this formulation is referred to as F18D. The saline-based aqueous F18 and F18D nanoformulations had a low viscosity, and were therefore not finalized for nasal delivery of EC16 due to rapid nasal mucociliary clearance mechanisms. To increase the viscosity, a commonly used food-grade thickening agent, carboxymethyl cellulose (CMC), was added to the nanoformulations. These mucoadhesive nanoformulations are referred to as F18C and F18DC, with 1% CMC (15 centipoise).

### 2.4. Evaluation of Particle Size Distribution

ZetaView nanoparticle tracking analysis was performed according to a method described previously [23,24]. The particle size distribution and concentration were measured using the ZetaView x20 (Particle Metrix, Meerbusch, Germany) and corresponding software. The measuring range for particle diameter is 10–2000 nm. These samples were diluted by the same volume of 1×PBS and then loaded into the cell. Particle information was collected from the instrument at 11 different positions across the cell, with two cycles of readings. The standard operating procedure was set to a temperature of 23 °C, a sensitivity of 70, a frame rate of 30 frames per second, and a shutter speed of 100. The post-acquisition parameters were set to a minimum brightness of 20, a maximum area of 1000, a minimum area of 10, and the trace length of 15 [24].

### 2.5. Direct Contact Antiviral Activity Tests

Infection of cells by OC43 virus, and viral titer: MRC-5 cells were cultured in EMEM Medium supplemented with 10% FBS and 1% penicillin, streptomycin, and amphotericin B. The viral infection assay and viral titering were performed in 96-well cell culture plates when the cells had reached 90% confluency. A 10-fold series dilution of OC43 virus in serum-free EMEM was loaded into wells in quadruplicate per dilution. After a 1-h absorption, the viral dilutions were removed and 100 µL serum-free EMEM was added, followed by incubation at 33 °C with 5% CO_2_ for >4 days to allow a CPE (cytopathic effect) to become visible. Viral titer was calculated by a TCID50 protocol and software [25]. A minimum of three independent experiments were performed and results recorded.

### 2.6. Electron Microscopy Imaging of the Viral Particles

The OC43 (log_10_ 8/mL) or 229E (log_10_ 6.7/mL) human coronavirus was incubated at a ratio of 1:9 with either saline (control) or F18D (1.25 mM EC16) for the indicated time period. Virus suspensions were then fixed in 4% paraformaldehyde and 2% glutaraldehyde by adding an equal volume of 8% paraformaldehyde and 4% glutaraldehyde to the viral mix, and 5 uL was transferred to a Formvar/Copper 200 mesh grid and allowed to dry for 15 min. Excess solution was then removed using filter paper and virus was negatively stained by addition of 5 uL of 2% aqueous uranyl acetate. Multiple images were captured from each sample in a JEM 1400Flash Transmission Electron Microscope (JEOL, Peabody, MA) at 120 kV, using a Gatan OneView Digital Camera (Gatan Inc., Pleasanton, CA, USA). The initial reaction ratio of OC43 viral particles to EC16 nanoparticles was 1:400; for 229E viral particles, the ratio was 1:60,000 before fixation.

### 2.7. Post-Infection Test

To test if EC16m nasal formulations possess a post-infection effect, human primary nasal epithelial cells (HNpECs) were allowed to form a monolayer (90% confluent) in a 24-well cell culture plate prior to a 1-h infection of OC43 virus in a series dilution to 10^−8^ before removal of virus suspension. Then, 500 µL of 5.7 mM EC16m (0.04%) in F18m nanoformulation were applied to one row of the wells for 10 min before being replaced by serum-free EMEM. The control wells were treated with airway basal medium only after viral infection before medium change to airway growth medium. Cytopathic effect (CPE) was captured after incubation for 8 days.

### 2.8. Cytotoxicity Assays

Human primary nasal epithelial cells (HNpECs) were allowed to form a monolayer prior to incubation with nanoformulations (F18m, F18Dm, F18C, F18DC) and control formulations (SG: saline and glycerol without EC16; D SG: dispersing agent, saline, and glycerol without EC16, or saline alone) in a series of dilutions for 60 min. The testing formulations were replaced with airway cell growth medium and incubated overnight. The MTT assay was performed the next day using CytoSelect MTT Cell Proliferation Assay kit (Cell Biolabs, Inc., San Diego, CA, USA) according to the method provided by the manufacturer.

### 2.9. Statistical Analysis

The primary statistical tests were parametric one-way ANOVA based on three or more repeated test points. The alpha was 0.05. GraphPad Prism version 6.0 software (www.graphpad.com) was used for most analyses. Reported errors are given as standard deviation (SD).

## 3. Results

### 3.1. Size Distribution and Zeta Potential of Particles

**Figure 1:**
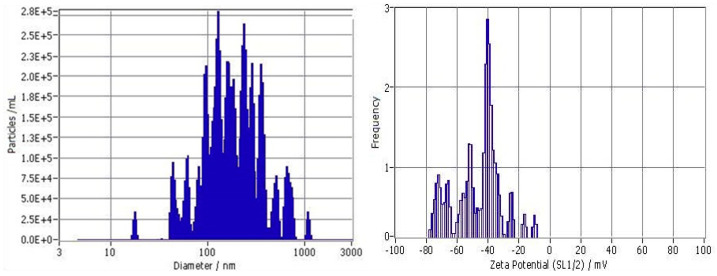
**Left:** Size distribution of particles in saline-diluted F18D. The size distribution profile for one representative sample determined by NTA is shown. **Right:** Zeta potential and distribution.

As shown in Figure 1, F18D showed a broad particle size distribution, with a median size of 186.6 ± 20.62 nm (SD, n = 3), and a cutoff size for 90% of the particles of <491 ± 92 nm (n = 3). There was only a slight difference in particle size distribution of F18D compared to our previously reported F18 nanoformulation [23]. The mean initial particle density was 4.57 × 10^7^/mL. The Zeta Potential of F18D diluted 30× by water at 25 °C is −50.24 ± 0.41 mV. Zeta Potential Distribution is 50.24 mV FWHM 5.15 (SL1/2). These particles appeared to be more evenly distributed in F18D compared to F18, with about 50% more particles/mL than F18 (45.7 × 10^6^ vs. 30.7 × 10^6^/mL with 0.001% EC16).

### 3.2. Contact Inhibition

**Figure 2:**
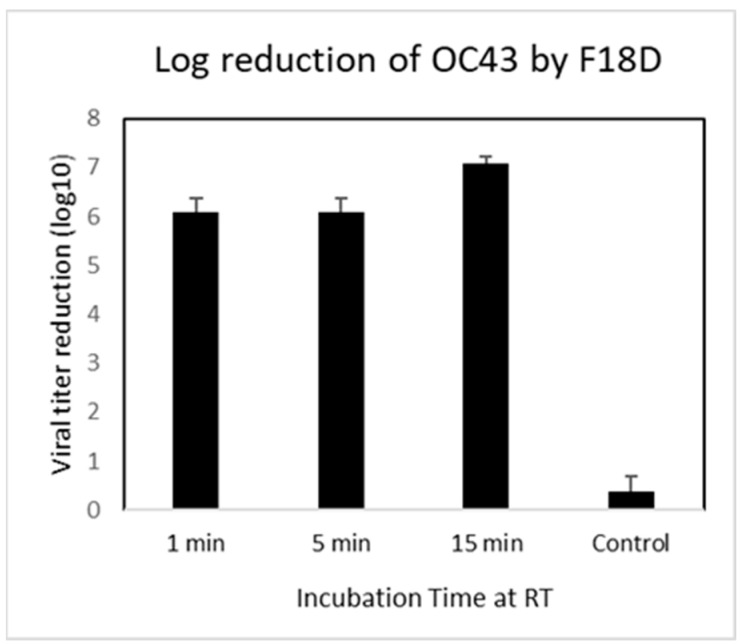
Time response of contact inhibition antiviral activity of F18D EC16 nasal formulation in saline containing 1.25 mM (0.1%) EC16 (n = 3) with an initial OC43 viral titer of log 7.75–9.5. This formulation was incubated with virus at a 1:9 ratio (virus to formulation) for 1, 5, and 15 min before 10× serial dilutions and TCID50 assay. The antiviral activity was calculated and expressed as log_10_ reduction ± standard deviation.

The time response of contact inhibition antiviral activity of the F18D EC16 nasal nanoformulation diluted with normal saline to a concentration of 1.25 mM EC16 (0.1%) was determined using MRC-5 cells. Direct contact incubation for 1 min (with gentle mixing) and 5 min both reduced viral infectivity by 6.08 ± 0.29 log_10_ (n = 3), while a 15-min incubation led to a 7.08 ± 0.14 log_10_ reduction (n = 3) (Figure 2). Separately, F18D reduced infectivity of 229E α-coronavirus by 4.58 ± 0.29 log_10_ after 30 min contact vs. control (F18D without EC16) of 0.167 ± 0.14 (n = 3, *p* < 0.0001), with a lower viral initial titer of 5.5 log_10_.

### 3.3. Transmission Electron Microscopy (TEM) Imaging of F18D-Treated Virus

**Figure 3:**
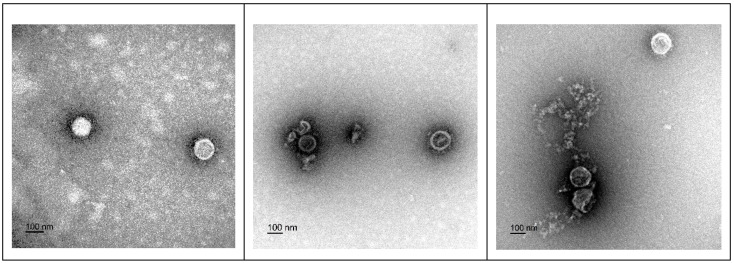
Representative original TEM images (from left to right) of saline incubated with 229E virus (1-min), 1-min exposure of OC43 (middle) and 229E virus (right) to the F18D EC16 nanoformulation. The saline-treated viruses show intact structure with a viral coat spike-like appearance. Both of the 1-min treated viral particles show structural changes and unknown debris/fragments, but there are viral particles that maintained apparently intact structures.

**Figure 4:**
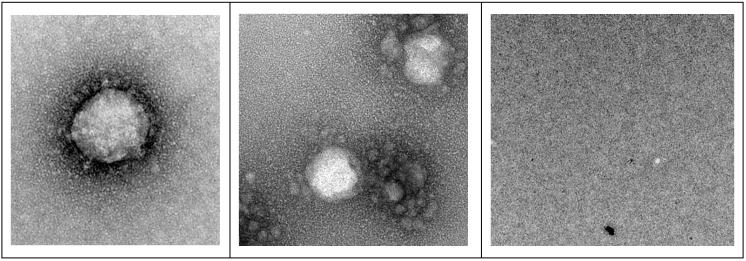
Representative TEM (enlarged) images of 30-min exposure of OC43 virus to Control (left, saline incubated virus) and virus treated with F18D EC16 nanoformulation (middle). The right image is a control of the nanoformulation without virus or saline. The saline-treated virus shows an intact structure with a viral coat spike-like appearance. The F18D EC16 nanoformulation-treated viral particles were smaller in size, and appeared to be associated with an unknown material.

Figure 3 shows representative images of virus samples treated for 1 min with saline (left), F18D/OC43 (middle), or F18D/229E (right). Aggregates of virus particles were not observed. Unlike the saline-treated 229E virus, both the OC43 and 229E virus treated with F18D demonstrated structural changes, with a loss of visible spikes (Figure 4), as well as the presence of smaller particles, often in aggregates. However, structurally recognizable OC43 and 229E viral particles were present after 1-min F18D treatment (Figure 3), suggesting the structural change effect was not universally immediate. This physical change mechanism of viral structure was also observed for the herpes simplex virus (Isaacs et al. [26]).

The results shown in Figure 4 demonstrated that saline failed to induce structural changes to the OC43 virus after 30 min incubation (left), while 30 min incubation with F18D significantly altered the appearance of viral particles (middle). There were approximately 2 × 10^9^ EC16 nanoparticles total in all of these fixed samples. However, there were no recognizable F18D nanoparticles in the images, suggesting 4% paraformaldehyde/2% glutaraldehyde fixation may have caused chemical dissociation of the nanoparticles. In summary, the TEM results showed a physical change to the virus particles, which correlated with the TCID50 results shown in Figure 2, in which 1-min contact to F18D resulted in a 6 log_10_ reduction in infectivity and longer incubation (15 min) lead to >7 log_10_ reduction.

### 3.4. Cytotoxicity towards HNpEC Cells Measured by MTT Cell Viability Assay

**Figure 5:**
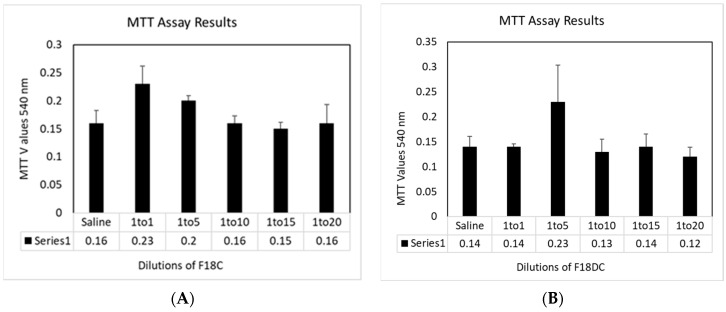
Cytotoxicity results from F18C ((**A**), F18 containing CMC) and F18DC ((**B**), F18D containing CMC) incubated with HNpECs for 60 min prior to the MMT assay.

The mucoadhesive nanoformulations are referred to as F18C and F18DC, with 1% CMC (15 centipoise). These formulations, as well as normal saline (carrier and treatment control), were diluted with airway cell basal medium and incubated with HNpECs for 60 min prior to overnight incubation and MTT assay. Figure 5A shows F18C diluted with basal medium at 1:1 (0.625 mM EC16) gave an MTT value of 0.23 ± 0.03, significantly higher than the saline control of 0.16 ± 0.02 (*p* = 0.038). When EC16 was diluted to 0.25 mM, the MTT value was also significantly higher than the saline control (0.20 ± 0.01, *p* = 0.048). When the EC16 concentrations were further diluted to 0.125, 0.008, and 0.063 mM, the MTT values are comparable to that of saline control without statistical difference (0.16 ± 0.02, *p* = 0.73; 0.15 ± 0.01, *p* = 0.51; 0.16 + 0.03, *p* = 1.0). A different pattern in MTT results was seen with F18DC.

As shown in Figure 5B, F18DC diluted with basal medium at 1:1 (0.625 mM EC16) gave an MTT value of 0.14 ± 0.01, similar to the saline control of 0.14 ± 0.02, *p* = 1.0. When EC16 was diluted to 0.25 mM, the MTT value was higher than the saline control but without statistical significance (0.23 ± 0.07, *p* = 0.12). When the EC16 concentration was further diluted to 0.125, the MTT value was lower than the saline control, but without statistical significance (0.13 ± 0.03, *p* = 0.43). At a concentration of 0.008 and 0.063 mM, the MTT values were comparable or lower than that of saline control, again without statistical difference (0.14 ± 0.03, *p* = 0.92; 0.12 ± 0.02, *p* = 0.25). Thus, the cytotoxicity of the nanoformulations F18C and F18DC was comparable to saline when the concentration range of EC16 is 0.0625 mM to 0.625 mM (0.005 to 0.05%).

### 3.5. Post-Infection Antiviral Activity of F18m in Nasal Epithelial Primary Cells (HNpECs, PromoCell)

**Figure 6:**
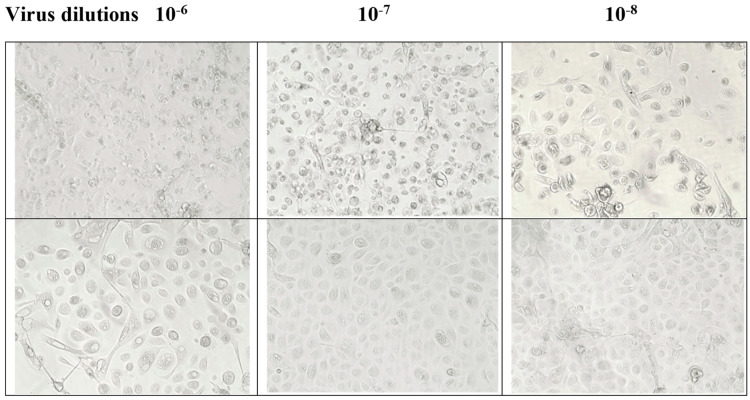
Representative images of HNpECs infected with OC43 in different dilutions (upper row) and the cells received a 10-min post-infection treatment of F18m at 50 μM (lower row). A cytopathic effect (CPE) was clearly shown in untreated cells infected with 10^−6^ and 10^−7^ dilutions, while F18m-treated cells did not show CPE. Photos were taken by an Evos XL Core microscope (200×) from Invitrogen Thermo Fisher Scientific, which lacks a scale bar function.

As shown in Figure 6, OC43-infected cells exhibited CPE in both 10^−6^ and 10^−7^ dilutions (upper row). The 10^−6^ dilution showed mostly dead cells, and the well at 10^−7^ dilution demonstrated typical cytopathic morphology, with a rounded cell shape and detachment from the surface. In contrast, F18m-treated wells are not associated with cell death or CPE morphology in the wells. These observations suggested that OC43 infects HNpECs and causes a cytopathic effect, whereas F18m-treated cells show no evident effect of viral infection.

### 3.6. Cytotoxicity Study of F18m and F18Dm in Normal Saline

**Figure 7:**
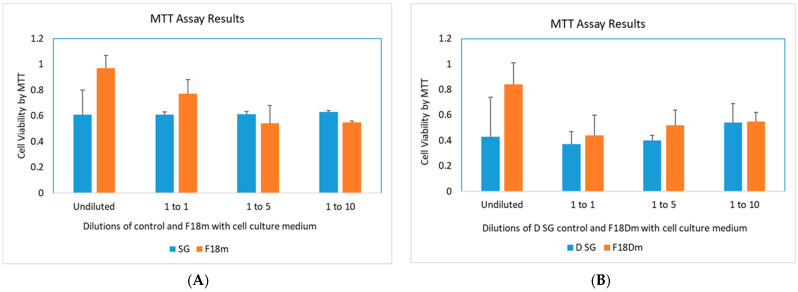
Cell viability results from MTT assays determined in human nasal primary epithelial cells (HNpECs) after incubation of the nanoformulations (orange bars) or control formulations (blue bars) for 60 min. (**A**). Results from the F18m nanoformulation containing saline, glycerol, and EC16m, and SG formulation containing saline and glycerol. (**B**). Results from the F18Dm nanoformulation containing saline, glycerol, EC16m and dispersing agent, and the D SG formulation containing dispersing agent, saline, and glycerol.

The MTT cell viability assay shown in Figure 7A indicates that undiluted 1.4 mM (0.1%) EC16m in F18m gave an MTT value of 0.97 ± 0.095, significantly higher than the SG control value of 0.61 ± 0.188 (one-way ANOVA, *p* = 0.04, n = 3) (Figure 5A). At a 1:1 dilution in saline (0.7 mM EC16m), there was no significant difference in MTT values between F18m and SG formulations (0.77 ± 0.11 vs. 0.61 ± 0.22, *p* = 3). When EC16m was diluted to 0.35 mM and 0.14 mM, there were small but significant decreases in MTT values compared to the control (0.55 ± 0.01 vs. 0.61 ± 0.02, *p* = 0.009; and 0.55 ± 0.01 vs. 0.63 ± 0.01, *p* = 0.001).

For the F18Dm nanoformulation, the results shown in Figure 7B indicated that undiluted (1.4 mM EC16m) F18Dm gave an MTT value of 0.84 ± 0.17, higher than the D SG control value of 0.43 ± 0.31 but without statistical significance (one-way ANOVA, *p* = 0.12, n = 3). At a 1:1 dilution (0.7 mM EC16m), there was no significant difference in MTT values between F18Dm and D SG formulations (0.44 ± 0.16 vs. 0.37 *+* 0.10, *p* = 0.57). When EC16m was diluted to 0.35 mM and 0.14 mM, there was no statistical difference in MTT values compared to the control (0.52 ± 0.12 vs. 0.40 ± 0.04, *p* = 0.17; and 0.55 ± 0.07 vs. 0.54 ± 0.15, *p* = 1.0). The untreated cells gave an MTT value of 0.59 ± 0.07.

In summary, the cytotoxicity of the nanoformulations F18m and F18Dm tested was comparable to the control formulations without EC16m. The concentration range of EC16m 0.14 mM to 1.4 mM (0.01 to 0.1%).

## 4. Discussion

The long-term goal of our studies is to develop an aqueous mucoadhesive nasal nanoformulation of EC16m with potent antiviral activity to minimize Long COVID neurologic symptoms. Due to the lipid-soluble nature of EC16, the solubility of EC16 is very low in aqueous solutions. By using our patent-pending technology, we were able to formulate glycerol-based nanoparticles comprised of EC16/EC16m into aqueous suspensions with normal saline, with particles ranging mostly in the nanometer range. This nanoformulation allows the lipid-soluble EC16/EC16m to be delivered to the cell surface without other nanoparticle-forming chemicals, encapsulation, or metals. From more than 100 formulations tested, the mechanically made F18/F18m glycerol-based stock nanoformulation showed the most promising results for its antiviral activity, with the simplest composition. The F18 and F18m nanoformulations were tested for antiviral activity with promising results [23]. The 18D nanoformulation is derived from F18 but includes a food-grade dispersing agent.

A crucial quality of nose-to-brain drug delivery (NTBDD) is the efficiency of targeted drug delivery. Results from ZetaView measurement of the size distribution of particles demonstrated the F18D nanoformulation had approximately 4.57 × 10^9^/mL for 0.1% (1.25 mM) EC16, and the median particle size was 186.6 + 20.62 nm (Figure 1). Previous studies suggest that a particle size around 200 nm would be suitable for nasal drug delivery [27]. Thus, the F18-based nanoformulations are appropriate for intranasal applications. The F18D nanoformulation showed a 50% increase in the number of particles compared to the F18 nanoformulation (from approximately 3 to 4.5 × 10^9^ particles/mL) [23].

The addition of the food-grade dispersing agent (proprietary, patent pending) not only increased the particle number, potentially by preventing aggregation, but it also increased the antiviral activity in comparison to the identical EC16 concentration alone (1.25 mM or 0.1%), and by a much greater fold than the increase in particle density. Figure 2 demonstrates that a 1-min viral contact with the F18D nanoformulation reduced the infectivity of OC43 virus by 99.9999% (6 log_10_). This rapid inactivation of OC43 (and 229E) virus was correlated with viral structural alterations shown in TEM imaging (Figure 3), which demonstrates rapid changes occurred in viral structure, while some viral particles appeared intact. When F18D was incubated with OC43 virus for 15 min, it reduced the viral infectivity by >99.99999% (7 log_10_) as shown in Figure 2. The structural changes in OC43 viral particles became more extensive after 30-min incubation with F18D, when intact viral particles were seldom seen in the fields (Figure 4). These results indicated that despite the F18 nanoformulation having acceptable high potency against human coronavirus (23), the efficiency of the nanoparticles can be further improved. That is, the F18D has passed the “antiviral” category to a “virucidal” category.

To determine if F18 and F18D were potentially cytotoxic, cell viability MTT assays were performed using the two nanoformulations in initial mucoadhesive formulations F18C and F18DC. Interestingly, F18C with an EC16 at 0.02 and 0.05% (0.25 and 0.625 mM) have higher cell viabilities than the saline control after 60 min-incubation with HNpECs (Figure 5A). This observation suggests EC16 at higher concentrations may protect the cells, or may trigger a higher mitochondrial activity. On the other hand, this EC16 concentration effect was not seen in F18D nanoformulations (Figure 5B), which requires more study on the dispersing agent. However, the results suggest that the thickening agent CMC is suitable for formulation optimization.

To further investigate the efficacy of the drug-grade EC16, EC16m was tested as an F18m nanoformulation in HNpECs to validate the previously obtained antiviral activity performed in MRC-5 cells [23]. The result shown in Figure 6 demonstrates a single 10-min post-infection incubation of F18m with 5.7 mM EC16m (0.04%) reduced OC43 viral replication by 2 log_10_ (99%). This result is consistent with the previously reported data, where F18m at a 12.5 to 50 μM range reduced OC43 viral replication in MRC-5 cells by >2 log_10_ (23).

The cytotoxicity of F18m and F18Dm was tested in HNpECs in comparison to the control without EC16m. A pattern found in F18 was also observed from F18m; higher EC16m concentrations were associated with higher cell MTT activity (Figure 7A). Both undiluted and 1:1 diluted (1.4 mM and 0.7 mM, or 0.1 and 0.05%) F18m have significantly higher MTT values than the lower concentrations, while the undiluted F18m has a significantly higher MTT value than the control. In contrast, the cell viability of F18Dm with 1.4 mM EC16m is not significantly higher than the control, but higher than other EC16m concentrations (Figure 7B). The major differences between the two MTT assays are the controls used. Results shown in Figure 5 are generated from a control of saline, while formulations without EC16m were used as controls for F18m and F18Dm.

The cytotoxicity data demonstrate that nanoformulations of EC16 and EC16m do not exhibit a higher cytotoxicity than their controls. At higher concentrations of EC16 or EC16m (0.02 to 0.1%), the nanoformulations may exert a protective effect on HNpECs, which could be due to increased mitochondrial activity. The advantage of the EC16/EC16m saline-based aqueous nanoformulations is that all ingredients are known nontoxic materials (EC16/EC16m, glycerol, saline, and food additive dispersing agent), and normal saline (0.9% NaCl) is regularly used in nasal irrigation/spray solutions.

In summary, it is known that the root cause of Long COVID-associated OD is persistent viral presence (persistent infection), inflammation of olfactory epithelia, and apoptosis of the epithelial/neuronal cells [4]. The results of the current study demonstrated that EC16/EC16m in the saline-based nanoformulations was able to rapidly inactivate β-coronavirus OC43, the strain with high genome homology with SARS-CoV-2 [28] by direct contact (1 min), and to reduce cytopathic effect after a post-infection treatment (10 min). With the known anti-inflammatory, antioxidant, and neuroprotective properties, intranasally delivered EC16m could potentially not only terminate the “persistent infection” in the OE, but also inhibit local inflammation and apoptosis, thereby normalizing the olfactory function and inhibit inflammation in the CNS.

## 5. Conclusions

In conclusion, an EC16m (drug grade) nasal nanoformulation was found to be a suitable basis for a new intranasal drug to minimize Long COVID-associated anosmia and other neurologic symptoms, pending additional formulation optimization and safety studies, especially on mucociliary safety tests, prior to clinical trials and drug development.

## 6. Patents

PCT/US23/74377 Pending: Compositions and methods of minimizing Long COVID. Inventor: Stephen Hsu (2023).

## Data Availability

Data are contained within the article.
